# Appropriate parboiling steaming time at atmospheric pressure and variety to produce rice with weak digestive properties

**DOI:** 10.1002/fsn3.617

**Published:** 2018-03-08

**Authors:** Elvire V. Zohoun, Sali A. Ndindeng, Mohamed M. Soumanou, Erasmus N. Tang, Jude Bigoga, John Manful, Sidi Sanyang, Noel H. Akissoe, Koichi Futakuchi

**Affiliations:** ^1^ Unité de Recherche en Génie Enzymatique et Alimentaire Laboratoire d'Etude et de Recherche en Chimie Appliquée Ecole Polytechnique d'Abomey‐Calavi Cotonou Bénin; ^2^ Africa Rice Center Bouake Côte d'Ivoire; ^3^ Faculty of Science University of Yaoundé‐I Yaoundé Cameroon; ^4^ Ecole de Nutrition et Sciences Alimentaires Faculté des Sciences Agronomiques Université d'Abomey‐Calavi Cotonou Bénin

**Keywords:** digestibility, gelatinization, paddy, rice varieties, vapor exposure time

## Abstract

Consumers with diabetes mellitus have shown interest in products with low postprandial glucose. To produce rice for this group of consumers, the effect of parboiling steaming time (0, 5, 15, 25, 35, and 45 min) and variety (NERICA1, NERICA7, WITA4, and IR841) on resistant, damaged starch fractions and glycemic response in rats was investigated. Resistant and damaged starch fractions were influenced by variety and steaming time but this was not the case for glycemic index. Nonparboiled NERICA7 and NERICA7 steamed for 25 min recorded the highest (10.07%) and lowest (2.49%) resistant starch fraction, respectively. Resistant starch correlated negatively with protein and sodium and positively with lipids. Damaged starch was high for WITA4 steamed for 45 min (26.80%) and low for nonparboiled NERICA1 (6.59%). Damaged starch correlated positively with lipid content and negatively with ash and total starch content. NERICA7 steamed for 35 min recorded the lowest postprandial glucose level 30 min after feeding (0.16 g/L), while WITA4 steamed for 15, 25, and 35 min and nonparboiled NERICA7 recorded higher levels (0.76, 0.91, 0.84, and 0.76 g/L, respectively). NERICA7 steamed for 35 min recorded both low glycemic and weak digestive properties because the glycemic index was lowest 120 min and increased steadily up to 180 min after feeding. We conclude that the digestive properties of rice depend both on the intrinsic properties of the variety and the parboiling steaming time.

## 1 INTRODUCTION

Rice is far the most important source of food for humans as world demand is expected to hit 533 million tons in 2030 compared to 472 million tons in 2015 (Food and Agricultural Organization, 2003). Increasing rice consumption is prominent in sub‐Sahara Africa, and changes in lifestyle and rapid urbanization have been suggested as possible causes. Most families prefer rice over other indigenous foods because they consider it cheap, fast to cook and tasty. Rice consumers are, however, very sensitive to quality and are ready to pay higher prices for better quality (Akoa Etoa et al., 2016; Demont et al., 2012). Although most consumers appreciate rice based on appearance, aroma (of uncooked grains), cooking time, swelling capacity, texture, and aroma (of cooked grains), some for health concerns (diabetes, obesity, and metabolic syndrome) are more interested in its digestibility (Fuentes‐Zaragoza, Riquelme‐Navarrete, Sánchez‐Zapata, & Pérez‐Álvarez, 2010). The latter group of consumers are those who are cautious on controlling their blood glucose level mainly due to diabetes mellitus. The glycemic index, which is the postprandial incremental glycemic area after a test meal, expressed as a percentage of the corresponding area of an equivalent reference meal such as glucose or white bread is commonly used to classify foods based on their postprandial blood glucose response (Goñi, Garcia‐Alonso, & Saura‐Calixto, 1997; Jenkins et al., 1987). Rice is predominantly composed of starch, which is in turn made up of amylose (linear polymer of α‐d‐glucose units linked by α‐1, 4 glycosidic bonds) and amylopectin (branched polymer of α‐d‐glucose units linked by α‐1, 4 and α‐1, 6 glycosidic bonds). Food products whose starch are slowly digested or resistant to enzyme digestion are preferred by some groups of consumers due to their health‐promoting benefits (Fuentes‐Zaragoza et al., 2010). Therefore, understanding the factors that favor the development of foods resistant to digestion is gaining a lot of attention worldwide. Earlier attempts led to the concept of starch fractions among which are rapidly digestible starch, slowly digestible, resistant starch (enzyme kinetic rate), and damaged starch (starch structure). Resistant starch is generally divided into three types: type I is starch that is protected in the plant cell, type II is native starch granules found in uncooked starch, and type III is retrograded starch (Englyst, Kingman, & Cummings, 1992). Damaged starch, which is the amount of starch granules that are physically damaged may occur either during the milling or cooking process and is hydrolyzed quickly by enzymes (Marti, Seetharaman, & Pagani, 2010). The size and molecular structure of the starch granules, the amylose:amylopectin ratio, the presence of pores, nonstarchy substances such as proteins, lipids, and phospholipids on the surface of granules, and processing methods have been shown to influence starch digestion.

Several authors have shown that small starch granules hydrolyze faster than larger ones because small granules have a larger specific area (Capriles, Coelho, Guerra‐Matias, & Arêas, 2008; Gao, Wong, Lim, Henry, & Zhou, 2015; Kaur, Singh, McCarthy, & Singh, 2007; Tester, Qi, & Karkalas, 2006). Uncooked cereal starches have been reported to show more hydrolysis compared to tuber and legume starches and this has been attributed to the presence of pores on cereal starch granules which facilitates the penetration of enzymes (Dreher, Dreher, Berry, & Fleming, 1984). The presence of proteins and lipids on the surface of granules can influence enzyme‐binding activity by blocking enzyme active sites (Oates, 1997) and thus reducing starch digestion. In addition, protein–starch interactions may also reduce the rate of digestion. Hu, Zhao, Duan, Linlin, and Wu (2004) showed that starch hydrolysis tended to be quick and complete for low than for intermediate and high amylose rice but other authors have also indicated that rice varieties similar in amylose content differed in starch digestibility and glycemic response in humans (Panlasigui et al., 1991). Although raw starch higher in amylopectin hydrolyzes faster than that higher in amylose due to its larger surface area per molecule (Hoover & Zhou, 2003), Lehmann and Robin (2007) indicated that amylopectin packing affected starch hydrolysis with the B‐form being more resistant than the A‐form because the B‐form was made of longer helices that were more stable. Studies on the effect of phosphorus on digestibility have not been reported but phospholipids have been shown to limit starch hydrolysis through the formation of inclusion complexes with amylopectin and amylose (Ahmadi‐Abhari et al., 2013; Singh, Dartois, & Kaur, 2010).

Processing of starch that results in size reduction (grinding), gelatinization (cooking), and lose of granule integrity (extrusion cooking) increases starch digestion because these processes make it easier for enzymes to attack the starch granules (Marti et al., 2010; Singh et al., 2010). Starch retrogradation and resistant starch formation in rice which may occur during the storage of cooked rice under refrigerated conditions have been shown to reduce digestibility and glycemic index (Hu et al., 2004; Mishra, Monro, & Hedderley, 2008). Rice parboiling, which is the hydrothermal treatment of rice, results in partial gelatinization and retrogradation of starch during the steaming, drying, and cooling processes. Chung, Lim, and Lim (2006) indicated that the relative melting enthalpy of rice starch samples was positively correlated to resistant starch and that at similar melting enthalpy, partially gelatinized samples were more resistant to digestion than those retrograded. Larsen, Rasmussen, Rasmussen, and Alstrup (2000) showed that mildly (soaking at ambient water temperature (28–30°C) for approximately 36 hr, steaming of the soaked rice at atmospheric pressure for 25–30 min, followed by drying at ambient temperature) parboiled rice had no effect on glycemic index, whereas severely (soaked at 70–75°C for 4 hr, followed by steaming at 120°C and 1.5 bar (1.5 × 10^5^ Pa) for 12 min and then predried at 100°C for 3 min before final drying at room temperature.) parboiled rice reduced glycemic index by 30% relative to the nonparboiled sample. Previously, we showed that rice variety and parboiling steaming time at atmospheric pressure affected the physicochemical and nutritional properties of rice with the possibility of selecting specific varieties and steaming time to achieve desired outcomes. The apparent amylose, total starch, protein and lipid content of the rice varieties: NERICA1, NERICA7, WITA4, and IR841 after parboiling for 5, 15, 25, 35, and 45 min have been reported (Zohoun et al., 2018). However, studies on the effect of the above steaming times and varieties on resistant, damage starch fractions and how these treatments affect postprandial glucose level and digestibility have not been done although such studies will be useful for the identification of varieties and steaming time for the development of rice products that may be beneficial to people suffering from diabetes mellitus. In this study, the effect of parboiling steaming time at atmospheric pressure and variety on resistant starch, damaged starch, and postprandial glycemic response in rats is reported.

## 2 MATERIALS AND METHODS

### 2.1 Rice varieties and grain production

Briefly, two upland NERICA rice varieties (1 and 7) and two sativa (irrigated) varieties (IR841 and WITA4) were obtained from the Genetic Resource Unit at AfricaRice and used for this study. The rice was planted in demonstration plots at AfricaRice station in Cotonou, Benin, in May and harvested in August 2014 using recommended agronomic practices (Saito et al. 2013; Tanaka et al. 2015). Briefly, the fields were double harrowed and well‐leveled before planting. For upland varieties, the seeding rate was 2–3 seeds per hill. Three weeks after planting, thinning was done to achieve 1 plant/hill. For irrigated varieties, plants from the nursery were transplanted at a rate of 1 plant/hill. The planting distance for both production systems was 25 by 25 cm, and 100 kg/ha nitrogen phosphorus potassium was applied before planting. Urea (25 kg/ha) was applied after first weeding and second weeding making a total of 50 kg/ha. Harvesting was done when the grain moisture was 20%–22% and dried to a moisture of 14% before storage.

### 2.2 Parboiling

Parboiling was done using the Grain quality enhancer, Energy‐efficient and durable Material (GEM) parboiler and procedures (Ndindeng et al., 2015). Briefly, 60 kg paddy of each cultivar was cleaned by winnowing and washing with clean water several times to remove impurities (poorly filled grains, rice plant debris, weed seeds, and soil). The washed paddy was transferred to the GEM soaking tank that was placed on an improved stove and water added to submerge the paddy (15 cm below the water surface). The fire was started, and the temperature of the water used for soaking was monitored until it recorded 85°C. At this temperature (initial soaking temperature), the GEM soaking tank with its contents was taken off the fire using a chain hoist system developed to reduce drudgery. The setup was left overnight at ambient conditions for 16 hr (soaking time) during which the temperature dropped gradually. The soaked paddy was drained, divided into five equal portions, and steamed for the following preset steaming times: 5, 15, 25, 35, and 45 min based on previous studies (Zohoun et al., 2018). Steaming was done in GEM steaming basket that allows only vapor generated in the tank to contact the soaked paddy. The water in the tank started to produce steam before the soaked paddy in the steaming basket was introduced into the tank, and the tank closed with a lid and this was when the recording of steaming time started. Steaming was terminated at the preset steaming time by immediately removing the paddy from the steaming pot. Parboiling experiments were replicated twice.

### 2.3 Drying

Steamed paddy was evenly sun‐dried on labeled tarpaulins placed on raised cemented surfaces and turned every 30 min. The moisture content of the grains was monitored during drying in a single kernel moisture tester (Kett model, PQ‐510). Sun drying was halted when the moisture content was 16%–18% and drying continued in the shade to final moisture content of 14% to avoid rapid drying at this small moisture range that could lead to fissures in the rice grain and increase breakages during milling (Bhattacharya, 1969).

### 2.4 Milling

Rice samples were dehusked using a large‐scale AGRINDO^®^ Rice Huller (P.T. Agrindo, Driyorejo, Indonesia) and polished using a large‐scale SB10D rubber roll mill (Satake‐Corporation, Hiroshima, Japan).

### 2.5 Preparation of rice flour

For each sample, 5 g of grains was ground to fine powder in a grinder (UDY cyclone mill; Fort Collins, Co., USA) fitted with a fine sieve of 0.5‐mm mesh size. The prepared rice flour was used for starch fraction analysis.

### 2.6 Starch fractions

#### 2.6.1 Resistant starch

Resistant starch was enzymatically determined using the Megazyme Resistant Starch Assay (K‐RSTAR, Megazyme Int. Co., Wicklow, Ireland) protocol (AOAC method 2002.02 and AACC method 32‐40.01). Briefly, 100 mg rice flour sample was hydrolyzed and solubilized in 4.0 ml of 10 mg/ml of pancreatic alpha‐amylase containing 3 U/ml of amyloglucosidase by incubating overnight (16 hr) at 37°C in a shaking water bath (Belco, Inc., USA) set at 100 revolutions/min. Ethanol (4.0 ml, 99% v/v) was added with vigorous stirring on a vortex mixer followed by a series of centrifugations at 2,000 g for 10 min to recover the resistant starch as pellets. The recovered resistant starch pellets were dissolved in KOH (2 ml, 2 mol/L) by stirring on an ice water bath for 20 min using a magnetic stirrer. Sodium acetate buffer (8.0 ml, 1.2 mol/L, pH = 3.8) was added with continuous magnetic stirring and immediately followed by the addition of amyloglucosidase (0.1 ml, 3,300 U/ml). This mixture was homogenized and incubated in a water bath at 50°C for 30 min with intermittent vortexing. Thereafter, the entire solution was transferred into a 100‐ml volumetric flask and volume adjusted to 100 ml with distilled water. An aliquot (3 ml) of this diluted solution was centrifuged at 2,000 g for 10 min. Duplicate 0.1 ml aliquots of the supernatant was transferred into clean tube, GOPOD reagent (3.0 ml) added, and the mixture incubated at 50°C for 10 min. The absorbance was read at 510 nm against the reagent blank constituted of sodium acetate buffer (0.1 ml, 100 mmol/L, pH = 4.5) and GOPOD reagent (3 ml). Resistant starch (g/100 g “as is”) was then computed using the Megazyme Mega‐Calc^™^ sheet for resistant starch (https://secure.megazyme.com/files/Data_Calculator/K-RSTAR_CALC.xls).

#### 2.6.2 Damaged starch

The measurement of damaged starch was done with the Megazyme starch damage assay kit (K‐SDAM, Megazyme Int. Co., Wicklow, Ireland) protocol (American Association of Cereal Chemist (AACC) approved method 76‐32.01 and ICC Method No. 164). Briefly, rice flour sample (100 mg) was weighed into glass test tube, pre‐equilibrated by adding fungal alpha‐amylase solution (50 U/ml), and incubated for 5 min in a water bath (Belco, Inc., USA) set at 40°C. Following equilibration, 1 ml of the fungal alpha‐amylase solution was added to the rice flour and vortexed for 5 s and then incubated at 40°C for 10 min. Eight milliliter sulfuric acid (2% v/v) was added at the 10th min and vortexed vigorously for 5 s to completely inhibit α‐amylase activity. The content of the tube was then centrifuged at 2,000 g for 10 min and 0.1 ml aliquots of the supernatant pipetted in duplicates into a clean test tube. Amyloglucosidase (0.1 ml, 2 U) was added into the tube, vortexed and incubated at 40°C for 10 min to complete the digestion of the disaccharides and trioses to monosaccharides. GOPOD reagent solution (4 ml) was then added into the tubes and into the glucose standard tube and further incubated for 20 min for color development. The absorbance was then read against a blank at 510 nm and the damaged starch of the sample computed using the Megazyme Mega‐Calc^™^ calculation sheet for starch damage (https://secure.megazyme.com/files/Data_Calculator/K-SDAM_CALC.xls).

### 2.7 Postprandial glucose measurement

WITA4 and NERICA7 varieties were used for this study because their resistant starch fraction response to steaming time was completely opposed. Resistant starch increased with steaming time for WITA4 but decreased with NERICA7. Six feed formulations based on steaming time replicated twice were produced from each rice variety and used to feed the experimental rats. A total of six male Albino rats (*Ratus norvegicus albino*) were used for the study. The animals were 2 months old with an average weight of 239 ± 13.5 g. The study lasted for 7 weeks (1 week of adaption and 6 weeks of experimentation). The feed formulation was made by mixing 1 g of rice flour (per variety and per steaming time) with 4 ml of deionized water. The six different rice treatments for each variety were given to the six (repetition) animals using a completely randomized design with each animal receiving all the treatments at the end of the experimental period. After the animals were fed, retro‐orbital blood was collected after 0, 30, 90, and 180 min and blood glucose determined spectrophotometrically. Glycemic index was determined as previously described (Goñi et al., 1997; Jenkins et al., 1987) using a 3‐hr period with the nonparboiled WITA4 as the reference meal. The study was approved by the ethical committee of the University of Abomey‐Calavi, whose guidelines were respected during animal handling at the Laboratory of Cytogenetics, Institute of Applied Biomedical Sciences, University of Abomey‐Calavi, Benin.

### 2.8 Statistical analysis

Line plots of resistant, total, and damage starch fractions against steaming time for NERICA1, NERICA7, WITA4, and IR841 were prepared, while bar charts of glycemic index against time after feeding were produced for NERICA7 and WITA4. Multivariate regression analysis was used to study the effect of rice variety and steaming time on resistant starch fraction, damage starch fractions, plasma glucose 30 and 90 min after feeding. Two‐way interactions between variety and steaming time for the different dependent variable above were compared using Fisher's least significant difference multiple comparison test followed by ranking. The relationship between resistant starch, damage starch, apparent amylose, protein, lipids, and mineral contents was determined using Pearson correlations. The statistical program used for the analysis was XLSTAT^™^ Premium software for Windows^®^ version 19.5 (Addinsoft SARL, Paris, France). All analyses were done at 5% significance level.

## 3 RESULTS AND DISCUSSION

### 3.1 Starch fractions

#### 3.1.1 Resistant starch

**Figure 1:**
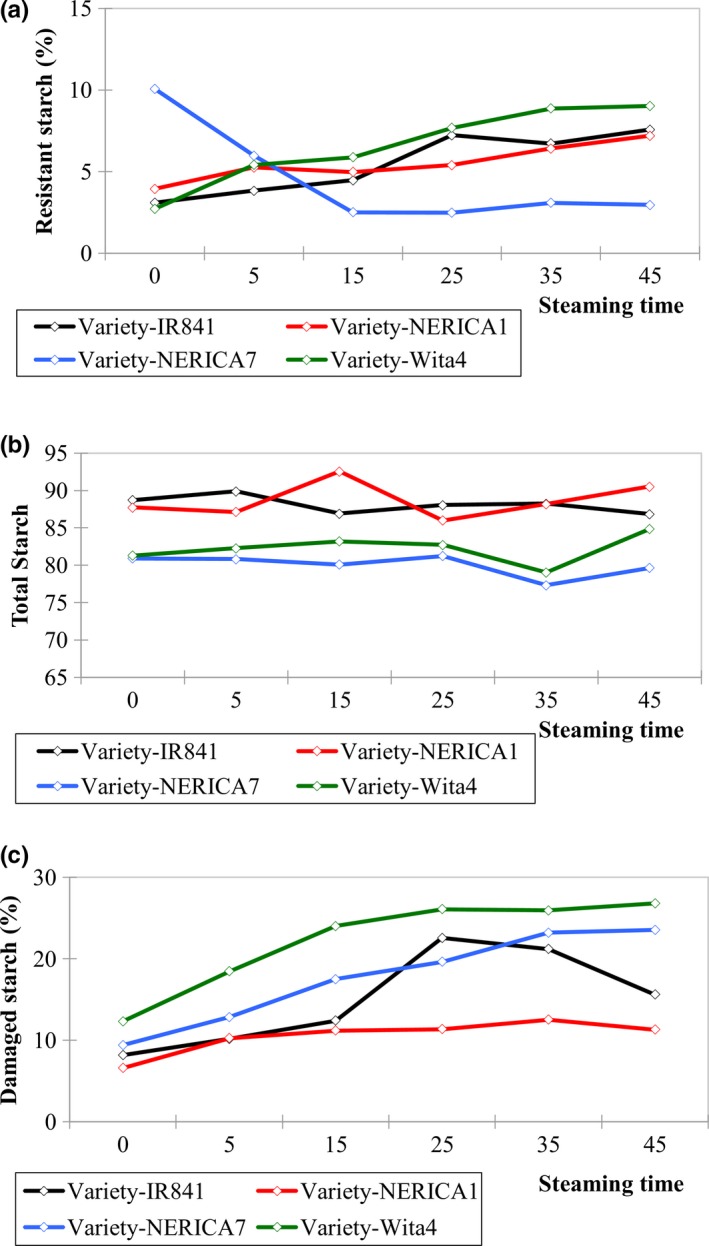
Effect of steaming time on (a) resistant starch, (b) total starch (c), and damaged starch fractions for some popular rice varieties grown in sub‐Sahara Africa

Resistant starch fraction was significantly influenced by steaming time and variety (*F* = 13.10, *p* < .0001) (Sup. 1). Nonparboiled NERICA7 and NERICA7 steamed for 25 min recorded the highest (10. 07%) and lowest (2.49%) resistant starch fraction, respectively. The amount of resistant starch present in NERICA7 steamed for 25 min was identical to that in NERICA7 steamed for 15 min and nonparboiled WITA4. Resistant starch increased with steaming time in three of the four varieties (NERICA1, IR841, and WITA4) (Figure 1a) probably due to crystallization of amylose after parboiling as previously reported (Eerlingen, Crombez, & Delcour, 1993; Mishra, Hardacre, & Monro, 2012) or amylose leaching during soaking and steaming (Patindol, Newton, & Wang, 2008). However, for NERICA7, higher amount of resistant starch in nonparboiled compared to parboiled samples indicated more crystallization in nonparboiled samples or the presence of a high amount of type II resistant starch (Englyst et al., 1992). It has been indicated that apparent amylose content was lower in parboiled than the nonparboiled counterpart due to amylose leaching during soaking and gelatinization during steaming (Patindol et al., 2008). Resistant starch correlated positively with apparent amylose content although not significantly (*R *= .19, *p* = .10) and amylose content decreased with steaming time as previously reported (Patindol et al., 2008; Zohoun et al., 2018). The rice varieties used in this study were either intermediate amylose or high amylose types (Table S1). NERICA1 and WITA4 recorded similar amounts of total starch, while the same was true for IR841 and NERICA7 (Figure 1b). The above observations suggest that the structure of the starch granules was more important for resistant starch formation in NERICA7, while starch retrogradation was more important for resistant starch formation for NERICA1, IR841, and WITA4.

#### 3.1.2 Damaged starch

Steaming time and variety influenced damaged starch fraction (*F* = 31.94; *p* < .0001) (Table S1). WITA4 steamed for 45 min recorded the highest damaged starch fraction (26.80%), while nonparboiled NERICA1 recorded the least (6.59%). Damaged starch increased with steaming time for all the rice varieties to 25 min and remained stable for three of the varieties while gently decreasing for IR841 (Figure 1c). Starch damage was highest in WITA4 and lowest in NERICA1 and this was true both for damage caused either by milling (steaming time = 0) or by parboiling (Steaming time = 5–45 min). WITA4 had the highest quantity of lipids, while NERICA1 had the least (Table S1). Damaged starch correlated positively with lipid content (*R* = .65; *p* < .0001) and ash content (*R* = .51, *p* < .0001) and negatively with sodium and total starch content (*R* = −.39; *p* < .05) (Table 1).

**Table 1:** Correlations between starch fractions and nutritional composition of NERICA1, NERICA7, IR841, and WITA4

Variables	Protein	Phosphorus	Potassium	Calcium	Magnesium	Sodium	Ash	Lipid	Total Starch	Resistant starch	Damaged Starch	Soluble starch
Protein												
Phosphorus	0.51^a^											
Potassium	0.42**	0.74^a^										
Calcium	0.06	0.43**	0.23*									
Magnesium	0.64^a^	0.86^a^	0.76^a^	0.38*								
Sodium	0.41**	0.32*	0.19	0.15	0.30*							
Ash	0.42**	0.50^a^	0.61^a^	−0.05	0.63^a^	−0.09						
Lipid	−0.32*	−0.26*	−0.08	−0.05	−0.14	−0.51^a^	0.18					
Total starch	−0.33*	−0.03	0.00	−0.12	−0.12	0.08	−0.31*	−0.33*				
Resistant starch	−0.24*	−0.16	−0.14	−0.04	−0.11	−0.28*	−0.14	0.32*	0.10			
Damaged starch	−0.06	0.07	0.21	−0.04	0.14	−0.39*	0.51^a^	0.65^a^	−0.39*	0.19		
Soluble starch	−0.21	0.03	0.06	−0.10	−0.06	0.20	−0.23*	−0.45^a^	0.90^a^	−0.32*	−0.45^a^	
Amylose content	−0.24*	−0.27*	−0.26*	−0.03	−0.27*	0.14	−0.59^a^	−0.04	0.36*	0.19	−0.26*	0.26*

a *p* < .0001; **p < .001; *p < .05.

#### 3.1.3 Postprandial glucose level, glycemic index, and digestibility

**Figure 2:**
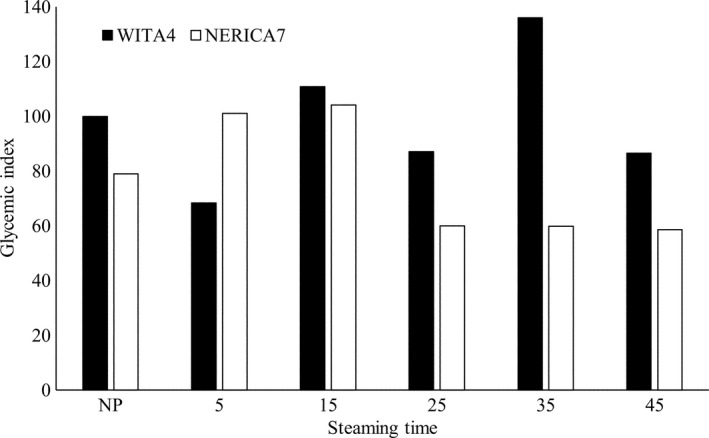
Glycemic index of WITA4 and NERICA7 parboiled at different steaming times recorded in rat 3 hr after feeding

Neither steaming time nor variety nor their interaction directly influenced postprandial glucose level and glycemic index in rats (*p* > .05) (Table 2). However, NERICA7 recorded lower glycemic indices compared to WITA4 during the 3‐hr period after feeding except for samples steamed for 5 min (Figure 2). This observation suggested that the starch in NERICA7 was more difficult to digest as compared to that of WITA4 except when steamed for 5 min. WITA4 was more digestible probably due to the higher quantity of damaged starch recorded compared to NERICA7 as observed by Singh et al. (2010). Zohoun et al. (2018) indicated that NERICA7 recorded high viscosities and still had some ungelatinized starch even after 45 min of steaming, whereas WITA4 was completely gelatinized. Our results are in conformity with Chung et al. (2006) who indicated that partially gelatinized samples were more resistant to digestion than those completely retrograded. NERICA7 steamed for 35 min recorded the lowest postprandial glucose level 30 min after feeding (0.16 g/L), while WITA4 steamed for 15, 25, and 35 min and nonparboiled NERICA7 recorded the highest (0.76, 0.91, 0.84, and 0.76 g/L, respectively) (*p* < .05). Nonparboiled NERICA7 recorded the lowest postprandial glucose level 90 min after feeding (0.15 g/L), while NERICA7 steamed for 5 min and WITA4 steamed for 35 min recorded the highest (0.54 g/L) (Table 2). Larsen et al. (2000) showed that severely parboiled rice reduced glycemic index by 30% relative to the nonparboiled sample. However, in this study, we did not see a direct link between the severity of parboiling and reduction in glycemic index, rather, specific varieties and parboiling regimes had to be identified to get the required effect on glycemic index. NERICA7 steamed for 35 min recorded low apparent amylose (high amylopectin), low total starch, and high protein content (Table S2 and S3). The low total starch and high protein in this sample suggest that a larger amount of starch granules in this sample were surrounded by a network of proteins and this could likely reduce their digestibility (Jenkins et al., 1987). Furthermore, the 35‐min steaming time was the time when the lowest total starch content was recorded for both WITA4 and NERICA7 suggesting that this steaming time provided the conditions for the starch granules in these varieties to undergo structural changes that reduced total starch available for quantification. These structural changes are probably linked to starch–protein interactions but this needs further investigation.

**Table 2:** Plasma glucose in rats 30 and 90 min after feeding as influenced by steaming NERICA7 and WITA4 at different parboiling times

Category	Plasma glucose 30 MAF (g/L)	Category	Plasma glucose 90 MAF (g/L)
WITA4*Steaming time‐25	0.91^a^*	Nerica‐7*Steaming‐5	0.54^a^
WITA4*Steaming time‐35	0.84^a^	Wita‐4*Steaming time‐35	0.54^a^
NERICA7*Steaming time‐0	0.76^a^	Nerica‐7*Steaming time‐15	0.48^ab^
WITA4*Steaming time‐15	0.76^a^	Wita‐4*Steaming time‐15	0.40^ab^
WITA4*Steaming time‐45	0.63^ab^	Wita‐4*Steaming time‐0	0.35^ab^
WITA4*Steaming time‐5	0.62^ab^	Nerica‐7*Steaming time‐35	0.35^ab^
NERICA7*Steaming time‐45	0.61^ab^	Nerica‐7*Steaming time‐45	0.33^ab^
WITA4*Steaming time‐0	0.58^ab^	Wita‐4*Steaming time‐45	0.29^ab^
NERICA7*Steaming time‐5	0.55^ab^	Wita‐4*Steaming time‐5	0.25^ab^
NERICA7*Steaming time‐15	0.52^ab^	Nerica‐7*Steaming time‐25	0.20^ab^
NERICA7*Steaming time‐25	0.45^ab^	Wita‐4*Steaming time‐25	0.17^ab^
NERICA7*Steaming time‐35	0.16^b^	Nerica‐7*Steaming time‐0	0.15^b^
Model goodness of fit	*R* ^2* *^ *= *.35; *f = *1.12; *P = *0.39		*R* ^2* *^ *= *.33; *F = *1.0; *P = *.42

MAF, minutes after feeding; Steaming time‐0: nonparboiled. *indicates that least square means with different letters are significantly different at 5% level

**Figure 3:**
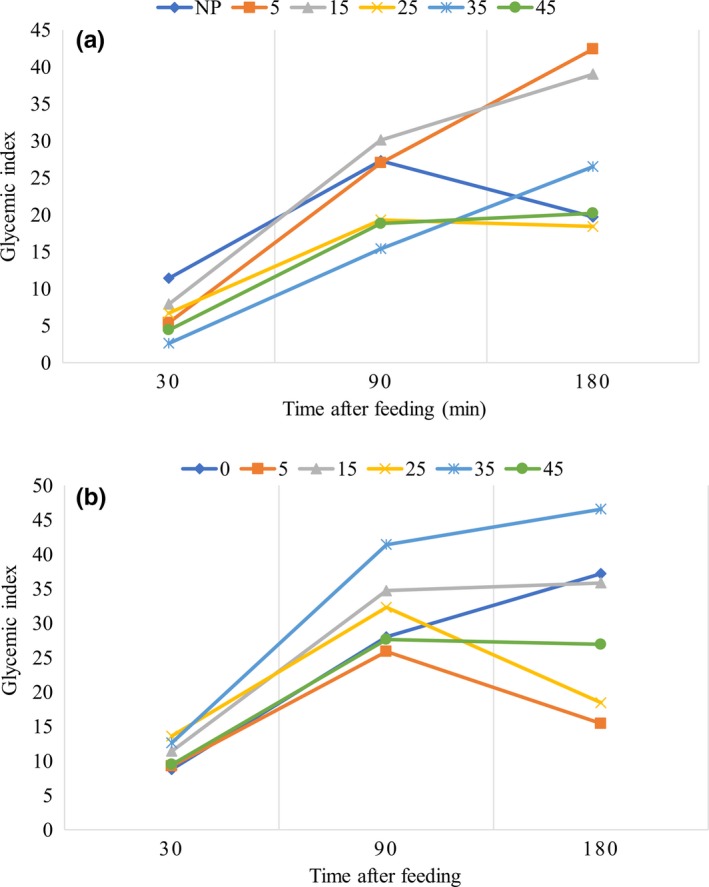
Glycemic and digestive properties of (a) NERICA7 and (b) WITA 4 parboiled using different steaming times

NERICA7 steamed for 35 min recorded both low glycemic and slow digesting properties because the glycemic index was lowest after 120 min and increased steadily up to 180 min after feeding (Figure 3a). Nonparboiled NERICA7 had a high glycemic index and was also rapidly digested. WITA4 steamed for 5 min recorded low digesting properties (Figure 3b) which was, however, higher than that recorded for NERICA7 steamed for 35 min. Differences in digestibility observed between the varieties as a function of steaming time indicate that specific rice varieties and parboiling regimes can be used to obtained end products with desired nutritional value such as low glycemic index.

## 4 CONCLUSION

Resistant and damaged starch fractions were influenced by variety and steaming time. Postprandial glycemic response was not directly affected by steaming time and variety. Resistant starch was higher in nonparboiled NERICA7 compared to the parboiled counterparts, whereas the opposite was true for NERICA1, WITA4, and IR841. The structure of starch granules (starch–protein interaction) was suggested as being important for resistant starch formation in NERICA7, while starch retrogradation was important for resistant starch formation in NERICA1, WITA4, and IR841. Reduced amylose played an important role in the formation of resistant starch in NERICA7, while proteins play a protective role in reducing starch damage in rice. The starch in NERICA7 was digested much slower in rats than that from WITA4 mainly because the starch in NERICA7 was not completely gelatinized even after steaming for 45 min. NERICA7 steamed for 35 min recorded the lowest postprandial glucose level 30 min after feeding (0.16 g/L) probably because this sample recorded low apparent amylose, total starch and high protein content facilitating the formation of starch granules that were resistant to digestion. In addition, NERICA7 steamed for 35 min recorded both low glycemic and weak digesting properties. Although this work reinforces the concept of selecting specific varieties and parboiling regimes to achieve desired processing outcomes, more work is needed to conform these results in humans and elucidate the structural characteristics of starch granules of the different parboiling treatments.
