# Supplementary material for: Appropriate parboiling steaming time at atmospheric pressure and variety to produce rice with weak digestive properties
Source: Food Sci Nutr. 2018 Mar 8;6(4):757–64. doi: 10.1002/fsn3.617 (PMC6021733; doi:10.1002/fsn3.617)
Supplement: Supplementary file 1 [file FSN3-6-757-s001.docx]

**Supporting file 1.** Effect of parboiling steaming time at atmospheric pressure and variety on protein, lipid, amylose and starch content in different rice varieties.

| Factors | *Category* | Protein (%) | Lipids (%) | Total Starch | Resistant starch (%) | Damaged Starch (%) | Amylose content (%) |
| --- | --- | --- | --- | --- | --- | --- | --- |
| Variety | *Wita4* | 9.20*^φ^* | 1.73 | 82.24 | 6.60 | 22.26 | 26.38 |
|  | *NERICA1* | 11.04 | 0.72 | 88.70 | 5.54 | 10.53 | 28.53 |
|  | *IR841* | 8.45 | 1.31 | 88.12 | 5.49 | 15.01 | 23.44 |
|  | *NERICA7* | 12.78 | 0.86 | 80.00 | 4.52 | 17.68 | 21.28 |
|  | *SD* | *1.94* | *0.46* | *4.31* | *0.85* | *4.91* | *3.19* |
|  |  |  |  |  |  |  |  |
| Steaming time (min) | *45* | 10.47 | 1.25 | 85.47 | 6.69 | 19.30 | 24.17 |
|  | *5* | 10.57 | 1.31 | 85.04 | 5.13 | 12.92 | 24.89 |
|  | *15* | 10.42 | 1.31 | 85.69 | 4.46 | 16.26 | 25.12 |
|  | *25* | 10.54 | 1.13 | 84.50 | 5.70 | 19.90 | 25.19 |
|  | *35* | 10.30 | 1.21 | 83.21 | 6.28 | 20.71 | 24.55 |
|  | *0* | 9.91 | 0.73 | 84.67 | 4.96 | 9.12 | 25.52 |
|  | *SD* | *0.24* | *0.22* | *0.89* | *0.84* | *4.57* | *0.49* |
|  |  |  |  |  |  |  |  |
| Two-way interactions | *Wita4*45* | 8.91 | 1.73 | 84.85 | 9.02 | 26.80 | 25.50 |
|  | *Wita4*25* | 9.52 | 1.61 | 82.74 | 7.68 | 26.06 | 27.20 |
|  | *Wita4*15* | 9.19 | 1.71 | 83.21 | 5.88 | 24.00 | 27.45 |
|  | *NERICA1*35* | 10.69 | 0.84 | 88.19 | 6.41 | 12.51 | 28.40 |
|  | *Wita4*35* | 8.91 | 1.83 | 79.04 | 8.87 | 25.93 | 25.50 |
|  | *NERICA1*45* | 11.06 | 0.70 | 90.55 | 7.20 | 11.29 | 28.52 |
|  | *NERICA1*5* | 11.62 | 1.13 | 87.15 | 5.26 | 10.26 | 28.83 |
|  | *Wita4*5* | 9.52 | 1.91 | 82.29 | 5.42 | 18.44 | 24.68 |
|  | *IR841*25* | 8.31 | 1.36 | 88.06 | 7.23 | 22.56 | 24.57 |
|  | *IR841*35* | 8.82 | 1.17 | 88.26 | 6.73 | 21.18 | 23.58 |
|  | *NERICA1*15* | 11.25 | 0.68 | 92.57 | 4.99 | 11.17 | 27.98 |
|  | *NERICA1*25* | 11.29 | 0.70 | 85.98 | 5.40 | 11.37 | 28.09 |
|  | *IR841*45* | 8.68 | 1.56 | 86.86 | 7.58 | 15.59 | 22.24 |
|  | *IR841*15* | 8.73 | 2.05 | 86.91 | 4.47 | 12.40 | 23.59 |
|  | *NERICA7*0* | 12.41 | 0.76 | 80.93 | 10.07 | 9.41 | 22.34 |
|  | *Wita4*0* | 9.15 | 1.61 | 81.30 | 2.72 | 12.33 | 27.93 |
|  | *NERICA1*0* | 10.31 | 0.29 | 87.76 | 3.95 | 6.59 | 29.36 |
|  | *NERICA7*5* | 12.74 | 0.71 | 80.81 | 5.98 | 12.83 | 21.85 |
|  | *NERICA7*45* | 13.21 | 1.03 | 79.64 | 2.96 | 23.54 | 20.41 |
|  | *IR841*5* | 8.40 | 1.48 | 89.91 | 3.83 | 10.15 | 24.20 |
|  | *NERICA7*25* | 13.02 | 0.87 | 81.23 | 2.49 | 19.60 | 20.91 |
|  | *NERICA7*35* | 12.79 | 1.00 | 77.34 | 3.10 | 23.21 | 20.71 |
|  | *NERICA7*15* | 12.51 | 0.81 | 80.08 | 2.51 | 17.49 | 21.48 |
|  | *IR841*0* | 7.75 | 0.25 | 88.71 | 3.10 | 8.18 | 22.44 |
|  | *SD* | *1.75* | *0.51* | *4.17* | *2.21* | *6.44* | *2.95* |

*φ: Value in red are highest while those in blue are lowest.*
